# Galunisertib attenuates pulmonary fibrosis with silicosis in mouse via TGF-β/TRAF6/Beclin1 signaling pathway

**DOI:** 10.3389/fphar.2025.1702511

**Published:** 2025-11-25

**Authors:** Rou Li, Huimin Kang, Aoxiang Hu, Guo Chen, Tinghua Yan, Ting Liu, Shi Chen

**Affiliations:** 1 Department of Psychiatry, The Second Xiangya Hospital, Central South University, Changsha, Hunan, China; 2 School of Public Health, Hunan Normal University, Changsha, Hunan, China

**Keywords:** Galunisertib, silicosis, TGF-β, autophagy, fibrosis

## Abstract

**Objective:**

Silicosis is characterized by silicon nodules and diffuse pulmonary fibrosis. To date, no effective therapy has been developed for the treatment of silicosis. This study aimed to investigate the effects of Galunisertib, a TGF-β receptor I kinase inhibitor, on the autophagy-lysosome system and pulmonary fibrosis in a SiO_2_-induced silicotic mouse model and cells.

**Methods:**

We established a silica-induced pulmonary fibrosis mouse and cell model. The MTT assay was used to determine the processing time and dose of cell experiments. Cell scratch assays were used to explore the effect of Galunisertib on the proliferation and migration ability of silica-stimulated fibroblasts. Cell migration was evaluated through wound healing, and the interactions between TGF-β and TRAF6/Beclin1 were verified by molecular docking and co-immunoprecipitation (Co-IP). WB and qPCR were used to detect the protein and transcription levels of TGF-β, Col-I, and α-SMA in each group, as well as the expression levels of autophagy-related protein LC3II/I, autophagy substrate protein p62, lysosome-associated membrane protein LAMP2, and pathway-related proteins TGF-β, TRAF6, and Beclin1. WB was also used to detect the expression level of apoptosis-related protein Cleaved-caspase 3 in the lung tissues and cells of mice in each group.

**Results:**

We found that Galunisertib has good anti-fibrosis activity both *in vitro* and *in vivo*. A 4-week Galunisertib treatment markedly ameliorated inflammation and fibrosis. Moreover, the results revealed that Galunisertib inhibited the expression of TGF-β, downregulated the major fibrotic protein expression of collagen I and a-smooth muscle actin (α-SMA), thereby switching the progression of fibroblast-to-myofibroblast transition (FMT). Furthermore, Evidence from Co-IP and molecular docking assays confirmed that this inhibition also involves the suppression of TRAF6 and Beclin1. Therefore, Galunisertib administration significantly altered the protein levels of LC3 and p62, implying that the autophagy-lysosome system might be involved in pulmonary fibrosis.

**Conclusion:**

These findings indicate that Galunisertib can modulate autophagy in pulmonary tissues of silicotic mice and fibroblast cells by suppressing the TGF-β/TRAF6/Beclin1 signaling pathway. On the other hand, Galunisertib regulates autophagy and inhibits the activation, proliferation and migration of Silica-stimulated fibroblasts, alleviating fibrosis in silicosis mice. Altogether, Galunisertib may be a potential candidate drug for preventing pulmonary fibrosis.

## 1 Introduction

Silicosis is a fibrotic occupational disease caused by prolonged and/or long-term inhalation of crystalline free silica (SiO_2_) dust and is characterized by diffuse inflammation and nodular pulmonary fibrosis in the lungs (Leung et al., 2012). Its pathological features include early infiltration of inflammatory cells, persistent pulmonary inflammation, excessive deposition of extracellular matrix (ECM), interstitial fibrosis, and formation of silicon nodules (Li S. et al., 2019). The presence of silicosis has been known for centuries; however, the pathological mechanism of silicosis is still unclear (Feng et al., 2020). Although lung transplant can help advanced patients extend life and improve quality of life, effective drugs for the treatment of silicosis are still lacking (Chu et al., 2019).

Although the etiology of silicosis is still unclear, emerging studies have shown that certain types of cells and cytokines play vital roles in the development of silicosis (Zhou et al., 2016). Fibrogenesis is a complex process that involves various factors, among which transforming growth factor-β (TGF-β) plays a pivotal role. It is widely accepted that TGF-β shows cytostatic effects in most epithelial cells, and TGF-β has been shown to inhibit proliferation of alveolar epithelial type II cells (Moses et al., 1990). TGF-β is also known as the most powerful inducer of epithelial-mesenchymal transition (EMT) (Saitoh, 2015). Since the discovery of TGF-β1, more than 30 TGF-β superfamily members have been identified and characterized, sharing common ground in synthesis, signal transduction mechanisms, and functions. Based on their similarity in structure and function, the TGF-β superfamily is divided into the TGF-β and bone morphogenetic protein (BMP) subfamilies. Typically, mammals express three homologous TGF-β subtypes (TGF-β1, TGF-β2, TGF-β3), among which TGF-β1 is most relevant to fibrosis.In fibrotic diseases, TGF-β1 is frequently upregulated and activated: it stimulates fibroblast proliferation and collagen production around silica particles—thereby promoting the development of pulmonary fibrosis and silicotic nodules—and triggers fibroblast-to-myofibroblast transition (FMT) to drive extracellular matrix (ECM) deposition (Wang et al., 2020). The activation of TGF-β signaling begins with the binding of the active TGF-β ligand to TGF-β receptor II (TβRII). This then forms a heterodimer with TβRI/ALK5. This complex then triggers the phosphorylation of intracellular proteins Smad2 and Smad3 at their C-terminus (Chaudhary et al., 2025). After being phosphorylated, Smad2 and Smad3 bind with Smad4 to form a complex that is then translocated to the nucleus to regulate the transcription of target genes, including Smad7. The latter is an inhibitory element that negatively regulates the activation and function of Smad2 and Smad3 (Hu et al., 2018). As a result, we hold the view that the regulation of the TGF-β1-Smad signaling pathway may be pivotal in the etiology of silicosis, although the exact mechanism remains indeterminate.

Galunisertib, also known as LY2157299 monohydrate, is an oral small-molecule kinase inhibitor that targets the TGF-β receptor type I (TGFβRІ). This agent has demonstrated that Galunisertib plays an important anti-fibrotic role in alleviating liver fibrosis, renal fibrosis, and cardiac fibrosis by attenuating the Smad and MAPK pathways and other pathways (Luangmonkong et al., 2017; Bigaeva et al., 2020; Nazari Soltan Ahmad et al., 2022). However, the role of Galunisertib in silicosis has not been reported. Therefore, in this study, we established a silica-induced pulmonary fibrosis mouse model and fibroblast model. We used both preventive and therapeutic administration strategies to investigate the therapeutic effect of Galunisertib on pulmonary fibrosis and explore its potential mechanisms. This will provide new ideas, methods, and a scientific basis for the treatment of clinical pulmonary fibrosis.

## 2 Materials and methods

### 2.1 Reagents and materials

Natural crystalline silica particles (Min-U-Sil 5 ground silica; size distribution: <5 μm, 97%) were obtained from the US Silica Company (Frederick, MD, United States); Galunisertib (700874-72-2, HPLC ≥98%) was purchased from Alfabiotech Company (Chengdu, China); antibodies against LC3B (2775), p62 (5114), and anti-K63-linkage Specific Polyubiquitin (D7A11) were obtained from Cell Signaling Technology (Danvers, Massachusetts, USA); Cleaved-caspase 3 antibody (AC033) was obtained from Beyotime Biotechnology (Shanghai, China); antibodies against Beclin1 (AF5128), Collagen I (Col-Ⅰ) (AF7001), Caspase 3 (AF6311), and glyceraldehyde-3-phosphate dehydrogenase (GAPDH) (AF7021) were obtained from Affinity Biosciences (Beijing, China); antibodies against TRAF6 (A23385), TGF-β (A21244), LAMP2 (A0593), α-smooth muscle actin (α-SMA) (A17910), HRP Goat Anti-Mouse lgG (AS003), and HRP Goat Anti-Rabbit lgG (AS014) were obtained from ABclonal (Wuhan, China).

### 2.2 Animals and treatments

Male C57BL/6J mice, aged 6–8 weeks and weighing 20–25 g, were obtained from the Hunan SJA Animal Co. Ltd (Certificate No. 2019-0004). The animals were housed in rearing cages of 45 cm × 35 cm × 20 cm, with 5 mice per cage. The temperature and relative humidity were kept at 21 °C–25 °C and 45%–65% respectively, and a 12-h light/12-h dark cycle was provided. The mice were supplied with standard laboratory mouse maintenance diet and sterile purified water *ad libitum*. The animals were acclimatized to the environment for 1 week before the experiment. The use of animals was approved by the Ethics Committees of Biomedicine Research, Hunan Normal University, Changsha, China (No. 2022460).

In this study, the mice were randomly divided into the following treatment groups: (1) Control (Ctrl) group, mice without any treatment; (2) crystalline silica (CS) group, mice received intratracheal injection of crystalline CS suspended in saline; (3) Galunisertib low dose group, mice received 100 µg/animal Galunisertib i.p.; and (4) Galunisertib high dose group, mice received 300 µg/animal Galunisertib i.p. (Supplementary Figure S1). The pretreatment of CS and the establishment of silicosis animal models were performed as previously described (Chen et al., 2015; Chen et al., 2021). Mouse lungs were collected 28/56 days after CS and Galunisertib administration.

### 2.3 Cell culture and treatments

MH-S (CL-0597) were kindly provided by Wuhan Pricella Biotechnology Co.,Ltd. L929 cells were donated by Professor Yang Fei’s research group at the University of South China. Galunisertib was dissolved in DMSO and sterile ultrapure water, respectively, which deliquated in culture medium to obtain a suitable working solution. MH-S cells were treated with 50 μg/cm^2^ silica, and L929 cells were incubated with Galunisertib, respectively. When the density reached about 80%, the silica suspension was added and incubated for 24 h. After centrifugation, the supernatant was used. When the L929 cells reached approximately 80% density, they were passaged and spread out in six-well plates. The following four groups were established: Control group, Silica group, Silica+5 μM Galunisertib group, and Silica+10 μM Galunisertib group. Corresponding treatments were applied to each group of cells, meaning they were treated with the supernatant of MH-S cells for 24 h. Then, the corresponding concentrations of Galunisertib were added to the Silica+5 μM Galunisertib group and Silica+10 μM Galunisertib group, respectively.

### 2.4 Measurement of pulmonary index

Mice were weighed after anesthesia, and their lung tissues were isolated and weighed after execution. The pulmonary index was calculated for each group of mice: Pulmonary index = Lung weight (g) × 100/Body weight (g).

### 2.5 Cell viability assay

Fibroblasts (1 × 10^4^ cells/well) were cultured in 96-well microtiter plates with serum-free DMEM for 24 h to induce growth arrest at 37 °C in a 5% CO_2_ incubator. Galunisertib was diluted in PBS and then filtered PBS was added to DMEM in each control group. The cells were incubated with different concentrations of Galunisertib (0,0.01,0.1,1,2,5,10, and 50 μM) for 24,48 or 72 h at 37 °C in a 5% CO_2_ incubator and treated for 4 h in medium containing 0.5% MTT 10 µL (at 37 °C). After removing the supernatant, 100 µL dimethyl sulfoxide was added to each well and the plates were mixed on a plate shaker for 10 min at room temperature. The absorbance of each sample was determined using a microplate reader at 570 nm.

### 2.6 Western blot

The pulmonary tissues or L929 cells were lysed using RIPA Lysis Buffer (P0013B, Beyotime, China) to extract the total protein. Nuclear and cytoplasmic proteins were separated from the pulmonary tissues or MH-S cells using Nuclear Protein Extraction Kit (R0050, Solarbio, China). Protein concentration was measured using BCA Protein Assay Kit (P0010, Beyotime, China). Denatured proteins were separated by electrophoresis on 8%-15% sodium dodecyl sulfate-polyacrylamide gels (SDS-PAGE) and transferred onto polyvinylidene fluoride (PVDF) membranes. The membranes were blocked with 5% non-fat milk and incubated with primary antibodies overnight at 4 °C. Then, the blots were incubated with horseradish peroxidase-conjugated secondary antibodies for 1 h at room temperature. Immunoreactive bands were detected using an enhanced chemiluminescence (ECL) kit (BL520A, Biosharp, China) followed by quantitative estimation using ImageJ software (National Institutes of Health, United States).

### 2.7 Quantitative PCR (qPCR)

Total RNA was extracted from lung tissues using TRIzol (R011-100, Genview, Beijing, China) according to the manufacturer’s protocol. Reverse transcription was performed using the Prime Script RT kit (RK20429, ABclonal, Wuhan, China), followed by qPCR using the SYBR Green Master Mix Kit (RK21204, ABclonal, Wuhan, China). The data were normalized to GAPDH as the endogenous control. Relative expression was calculated using the 2^−ΔΔCt^ method. The specific primer sequences were as follows: *Beclin1*, F: CTT​CAA​TGC​CAC​CTT​CCA, R: CTG​TCA​GAG​ACT​CCA​GAT​AG; *p62*, F: GCA​CAG​GCA​CAG​AAG​ACA​AG, R: CCA​CCG​ACT​CCA​AGG​CTA​TC; *LAMP2*, F: ACA​CTC​ACT​CCA​ACT​TCA​ACA​C, R: GGT​AGC​CAG​CAG​ACA​GGT​AG; *Caspase-3*, F: GCT​GAC​TTC​CTG​TAT​GCT​TAC​TC, R: AAT​TCC​GTT​GCC​ACC​TTC​CT; *Col-Ⅰ*, F: CAG​TGG​CGG​TTA​TGA​CTT​CAG, R: GGC​TGC​GGA​TGT​TCT​CAA​TC; *α-SMA*, F: GAA​CAC​GGC​ATC​ATC​ACC​AA, R: ATC​TCC​AGA​GTC​CAG​CAC​AAT​A; *TGF-β*, F: CTG​CTG​ACC​CCC​ACT​GAT​C, R: GGG​GCT​GAT​CCC​GTT​GAT​T, *TRAF6*, F: TGC​TTG​ATG​GCT​TTA​CGG​GA, R: TTG​TGC​CCT​GCA​TCC​CTT​ATG, *GAPDH*, F: AAT​GGT​GAA​GGT​CGG​TGT​GA, R: CGC​TCC​TGG​AAG​ATG​GTG​AT.

### 2.8 Pathohistological examination

The lungs of the mice were immersed using 4% neutral buffered formalin followed by paraffin embedding and cut into 5 μm sections. Then inflammation and fibrosis were assessed by hematoxylin and eosin (H&E) and Masson’s trichrome staining of paraffin lung sections, according to the manufacturer’s protocol. Fibrosis was scored using the ImageJ software (National Institutes of Health, USA). A mean value of the fibrosis scores was determined by evaluating three different fields of each section, with a total of three sections per animal (5 mice/group).

### 2.9 Measurement of HYP content

The levels of Hydroxyproline (HYP) in lung tissue samples were quantified using the assay kit (A030-2-1, Jiancheng, Nanjing, China) according to the manufacturer’s instructions. The absorbance was measured at 550 nm respectively. HYP content was calculated as μg/mg wet lung.

### 2.10 Co-immunoprecipitation (Co-IP)

Co-IP was performed using indicated antibodies and IgG (Invitrogen) according to manufacturer’s instruction. In brief, cell lysates were incubated with antibody-conjugated beads at 4 °C for 2 h. Then, the beads were washed extensively and boiled in SDS loading buffer. Western blot was used to study the immunoprecipitated proteins.

### 2.11 Construction of protein-protein interaction (PPI) network

The PPI network was constructed using the STRING database (https://cn.string-db.org/) and the GENEMANIA database (https://genemania.org/).

### 2.12 Molecular docking

PDB format files for the key genes were retrieved from the RCSB database (https://www.rcsb.org/), and molecular structure files for Galunisertib were downloaded from the PubChem database (https://pubchem.ncbi.nlm.nih.gov/). The docking simulations were then conducted using CB-Dock2 (Liu et al., 2022; Yang et al., 2022).

### 2.13 Statistical analysis

Measurement data were expressed as mean ± standard deviation (SD) and statistical significance was determined using one-way analysis of variance (ANOVA) and Student-Newman-Keuls test (for multiple groups). A value of *P* < 0.05 indicated statistical significance.

## 3 Results

### 3.1 Effect of Galunisertib on physiological and pathological indicators during the development of silicosis

**FIGURE 1:**
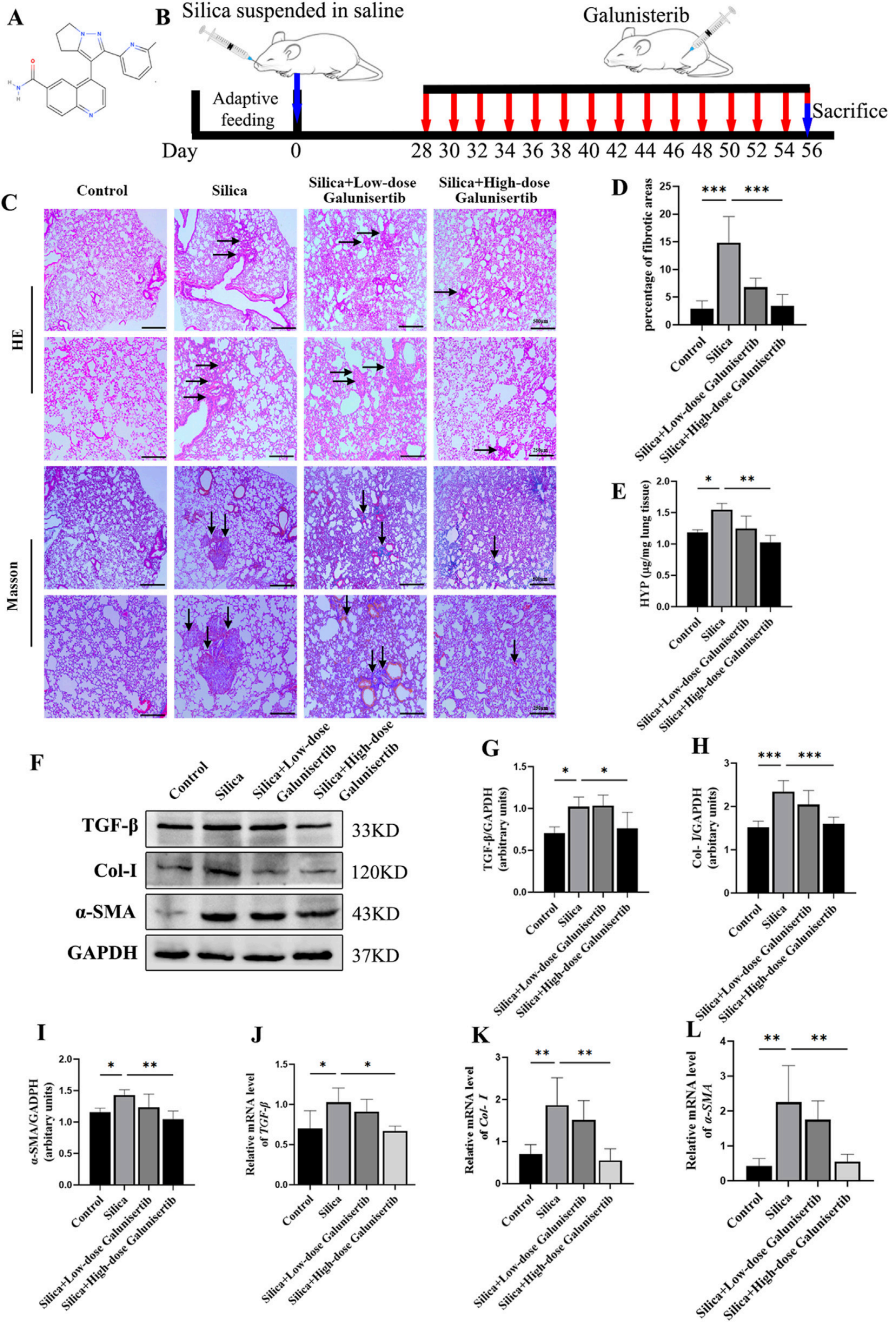
Effect of Galunisertib on physiological and pathological indicators during the development of silicosis. **(A)** The chemical structure of Galunisertib. **(B)** The timeline of animal experiments. **(C)** H&E and Masson staining of the mouse lung tissue (original magnification: upper images, ×50 and lower images, ×100). **(D)** The score analysis of Masson staining in the mouse lung tissue. **(E)** Quantification of collagen in the mouse lung tissue by HYP content. **(F–I)** Western blot detection of TGF-β, Col-Ⅰ and α-SMA of each group, with GAPDH as the loading control. **(J–L)** qPCR detection of *TGF-β*, *Col-I*, and *α-SMA* transcriptional levels in the pulmonary tissue of each group, with *GAPDH* as the loading control. Significance was determined using one way ANOVA. n = 3-5; The right arrows represent inflammatory cell infiltration and damaged alveolar structure, and the down arrows represent collagen deposition. ***, *P* < 0.001; **, *P* < 0.01; *, *P* < 0.05.

Galunisertib has been found to exhibit antifibrotic and antitumor efficacy at doses ranging from 40 mg to 300 mg every 2 days in a variety of organ fibrosis models and cancer patients. After the conversion and comprehensive consideration of these doses, two dose groups of 100 μg/mice and 300 μg/mice were preliminarily selected to intraperitoneally inject the mouse model every other day until sacrifice, so as to preliminarily explore the role of Galunisertib in the mouse model of silicosis and determine the optimal drug dose. To explore the role of Galunisertib in the formation of pulmonary fibrosis in silicosis mice, we established a silica-induced silicosis mouse model and injected silica-induced silicosis mice with different concentrations of Galunisertib for 4 weeks (Figure 1B). As shown in Figure 1C, after lung pathological dissection of mice after 56 days of dusting, we found that Galunisertib treatment alleviated silica-induced fibrotic lesions on the lung surface. The results of H&E and Masson staining showed that the alveolar structure was destroyed with inflammatory cell infiltration in the silica group, and several obvious fibrous nodules appeared, and the intervention of Galunisertib significantly alleviated the above pathological changes. The results of Masson staining also showed that a large amount of blue collagen deposition appeared in the lung tissue of the silica group, and the intervention of Galunisertib significantly reduced the collagen deposition (Figures 1C,D). In addition, the result also showed that Galunisertib inhibited silica-induced collagen deposition in the lungs (Figure 1E). Galunisertib, as a TGF-β inhibitor (Figure 1A), significantly reduced the protein and mRNA levels of TGF-β in silica-induced silicosis mice, and Western blot analysis showed that high-dose Galunisertib also significantly inhibited the expression of Col-I and α-SMA proteins, two important markers of fibrosis (Figures 1F–I). qPCR analysis of transcription levels of *Col-I* and *α-SMA* further demonstrated the inhibition of high-dose Galunisertib (Figures 1J–L). Therefore, we subsequently used high doses for *in vivo* experiments. In summary, these results provide *in vivo* evidence that high-dose Galunisertib can inhibit silica-related pulmonary fibrosis.

### 3.2 Galunisertib ameliorates silica-induced pulmonary fibrosis progression *in vivo* in therapeutic intervention regimens

**FIGURE 2:**
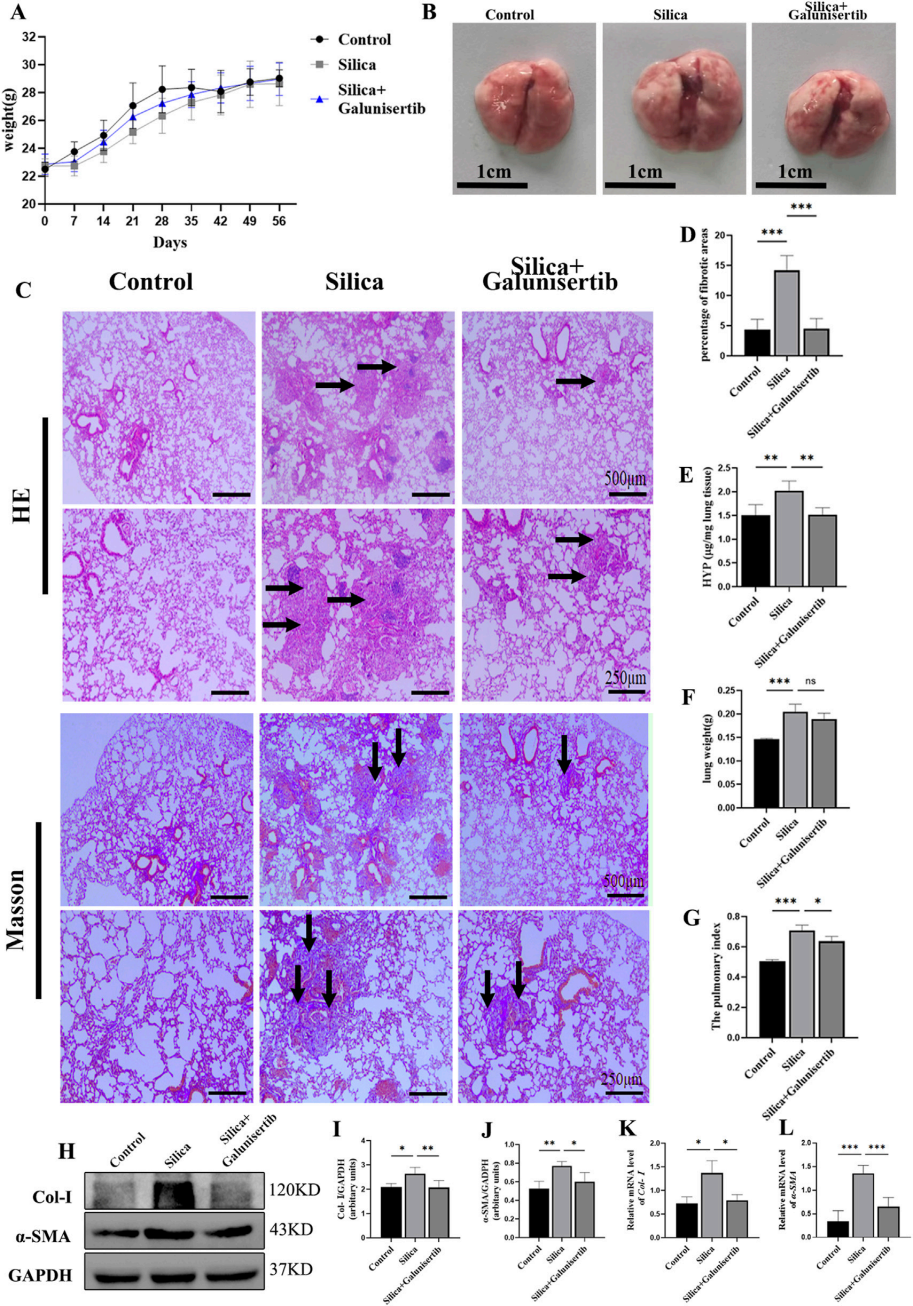
Galunisertib ameliorates silica-induced pulmonary fibrosis progression *in vivo* in therapeutic intervention regimens. **(A)** Body weights of experimental mice from indicated groups. **(B)** Representative images of lung tissue from each group of mice. **(C)** H&E and Masson staining of the mouse lung tissue (original magnification: upper images, ×50 and lower images, ×100). **(D)** The score analysis of Masson staining in the mouse lung tissue. **(E)** Quantification of collagen in the mouse lung tissue by HYP content. **(F)** Lung weights of mice in each group. **(G)** The pulmonary index was determined to show the alveolar swelling and interstitial fibrosis in mice. **(H–J)** Western blot detection of Col-Ⅰ and α-SMA of each group, with GAPDH as the loading control. **(K,L)** qPCR detection of *Col-I*, and *α-SMA* transcriptional levels in the pulmonary tissue of each group, with *GAPDH* as the loading control. Significance was determined using one way ANOVA. n = 3–5; The right arrows represent inflammatory cell infiltration and damaged alveolar structure, and the down arrows represent collagen deposition. ***, *P* < 0.001; **, *P* < 0.01; *, *P* < 0.05.

To test the antifibrotic efficacy of Galunisertib in a preclinical model of silicosis *in vivo*, we constructed two treatment models with different time stages (Supplementary Figures S1B,C). It was found that therapeutic administration of Galunisertib could alleviate silica-induced pulmonary fibrosis. The results of H&E and Masson staining showed that, compared with the control group, the lungs in the silica group presented round or oval-shaped silicon nodules of various sizes. In some areas, these nodules tended to form clusters. Macrophages, neutrophils infiltration, and fibroblast proliferation were observed in the silicon nodules. In contrast, the number of silicon nodules in the silica + Galunisertib group was significantly lower than that in the silica group, and the volume of the nodules was smaller. The area of blue collagen deposition was significantly reduced (Figures 2B–D). The hydroxyproline (HYP) content assay also indicated that therapeutic administration of Galunisertib could effectively inhibit silica-induced pulmonary collagen deposition (Figure 2E). Although there were no significant differences in the absolute body and lung weights of mice compared to the silica group, the lung weight/body weight ratio was significantly decreased. (Figures 2A–G). In addition, Western blot and qPCR analyses demonstrated that therapeutic administration of Galunisertib could significantly reduce the expression of fibrosis-related proteins: Col-Ⅰ and α-SMA (Figures 2H–L). However, these changes were not significant during the preventive treatment stage (Supplementary Figure S2). These results suggest that administration of Galunisertib during the treatment stage can effectively delay the progression of pulmonary fibrosis in silicosis mice.

### 3.3 Galunisertib mitigates silica-induced apoptosis in silicotic mice

**FIGURE 3:**
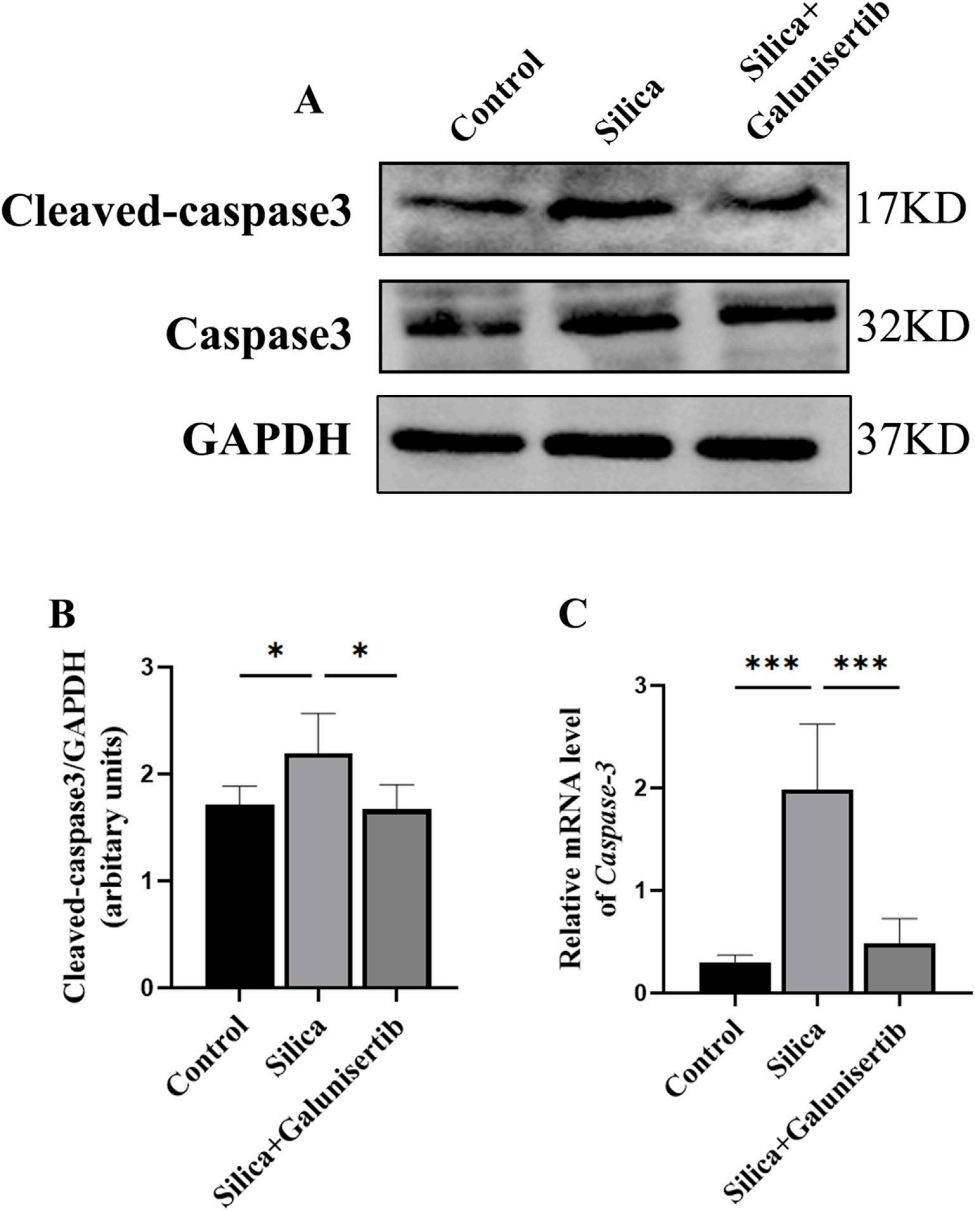
Galunisertib mitigates silica-induced apoptosis in silicotic mice. **(A,B)** Western blot detection of Cleaved-caspase 3 protein level in the pulmonary tissue of each group, with GAPDH as the loading control. **(C)** qPCR detection of *Caspase 3* transcriptional levels in the pulmonary tissue of each group, with *GAPDH* as the loading control. Significance was determined using one-way ANOVA. n = 3-5; ***, *P* < 0.001; *, *P* < 0.05.

Next, the effect of Galunisertib on pulmonary tissue apoptosis was investigated in silica-treated mice. Silica remarkably elevated the concentrations of Cleaved-caspase 3 in lung tissues (Figures 3A–C). The silica-induced release of pro-apoptosis cytokines was attenuated by Galunisertib treatment, indicating the anti-apoptosis function of Galunisertib (Figures 3A–C).

### 3.4 Galunisertib inhibits proliferation, migration, and FMT in silica-stimulated fibroblasts

**FIGURE 4:**
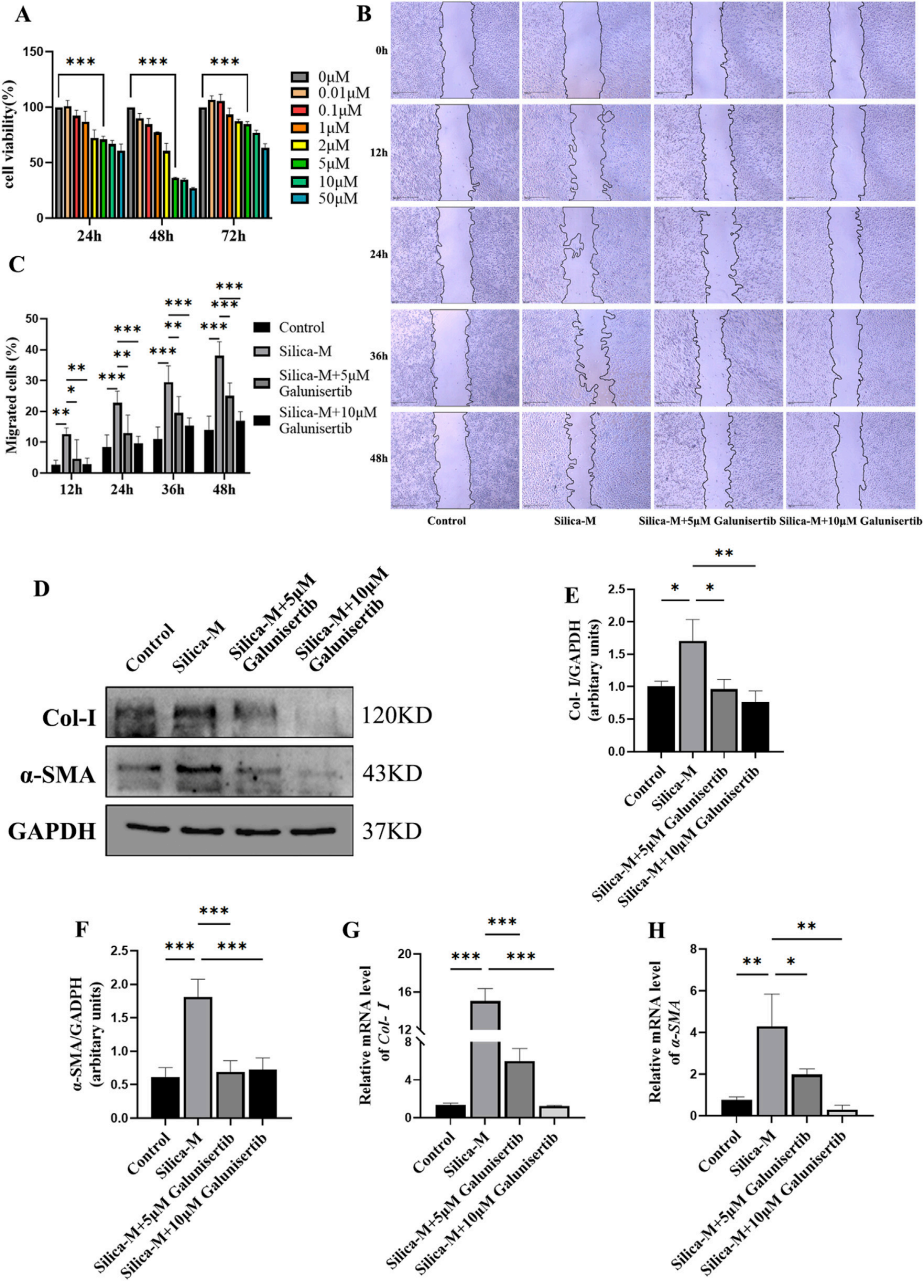
Galunisertib inhibits proliferation, migration, and FMT in Silica-stimulated fibroblasts. **(A)** The cell viability of fibroblasts was measured using MTT in response to various concentrations of Galunisertib treatment. **(B)** The impact of scratch assay on the proliferation and migration ability of fibroblasts treated with Galunisertib towards Silica was examined. **(C)** Statistical analysis of the impact of Galunisertib on the proliferation and migration ability of fibroblasts induced by Silica. **(D–F)** Western blot detection of Col-Ⅰ and α-SMA protein level in fibroblasts of each group, with GAPDH as the loading control. **(G,H)** qPCR detection of *Col-I* and *α-SMA* transcriptional levels in fibroblasts of each group, with *GAPDH* as the loading control. Significance was determined using one-way ANOVA. n = 3-5; ***, *P* < 0.001; **, *P* < 0.01; *, *P* < 0.05.

To evaluate the effect of Galunisertib on fibrosis *in vitro*, we determined the safe concentration of Galunisertib by MTT assay. The results showed that the application of Galunisertib with a concentration of 5 μM or more significantly inhibited cell viability at all three time points, indicating that drugs within this concentration range can produce definite biological effects on cells (Figure 4A). Therefore, 5μM and 10 μM of Galunisertib were chosen to explore the anti-fibrosis activity. Fibroblasts were divided into Control, Silica-M, Silica-M+5 μM Galunisertib, and Silica-M+10 μM Galunisertib groups. The fibroblast migration rate of Silica-M stimulated fibroblasts significantly increased after 12 h, 24 h, 36 h, and 48 h compared with that of the Control group. However, the migration rate of fibroblasts slowed down after the addition of Galunisertib for 12 h, 24 h, 36 h, and 48 h (Figure 4B). Quantitative analysis was performed and significant differences among the three groups were identified (Figure 4C). These results indicate that Galunisertib inhibits the migration and proliferation of Silica-M stimulated fibroblasts. Moreover, the results showed that the expression of Col-Ⅰ and α-SMA protein in fibroblasts significantly increased after Silica-M treatment (Figures 4D–F). Compared with the Silica-M group, the expression levels of Col-Ⅰ and α-SMA decreased after different concentrations of Galunisertib were added. In the qPCR experiment, we used the same group as that utilized for the Western blot experiment. After treatment with different concentrations of Galunisertib, Col-Ⅰ and α-SMA in the Silica-M group were significantly upregulated compared with the control group, whereas their expression levels in the Galunisertib groups were lower than those in the Silica-M group (Figures 4D,G,H). In conclusion, the results suggest that Galunisertib inhibits the expression of Col-Ⅰ and α-SMA of Silica-M stimulated fibroblasts, indicating that Galunisertib restrains the conversion of fibroblasts into myofibroblasts.

### 3.5 Analysis of potential targets of Galunisertib

**FIGURE 5:**
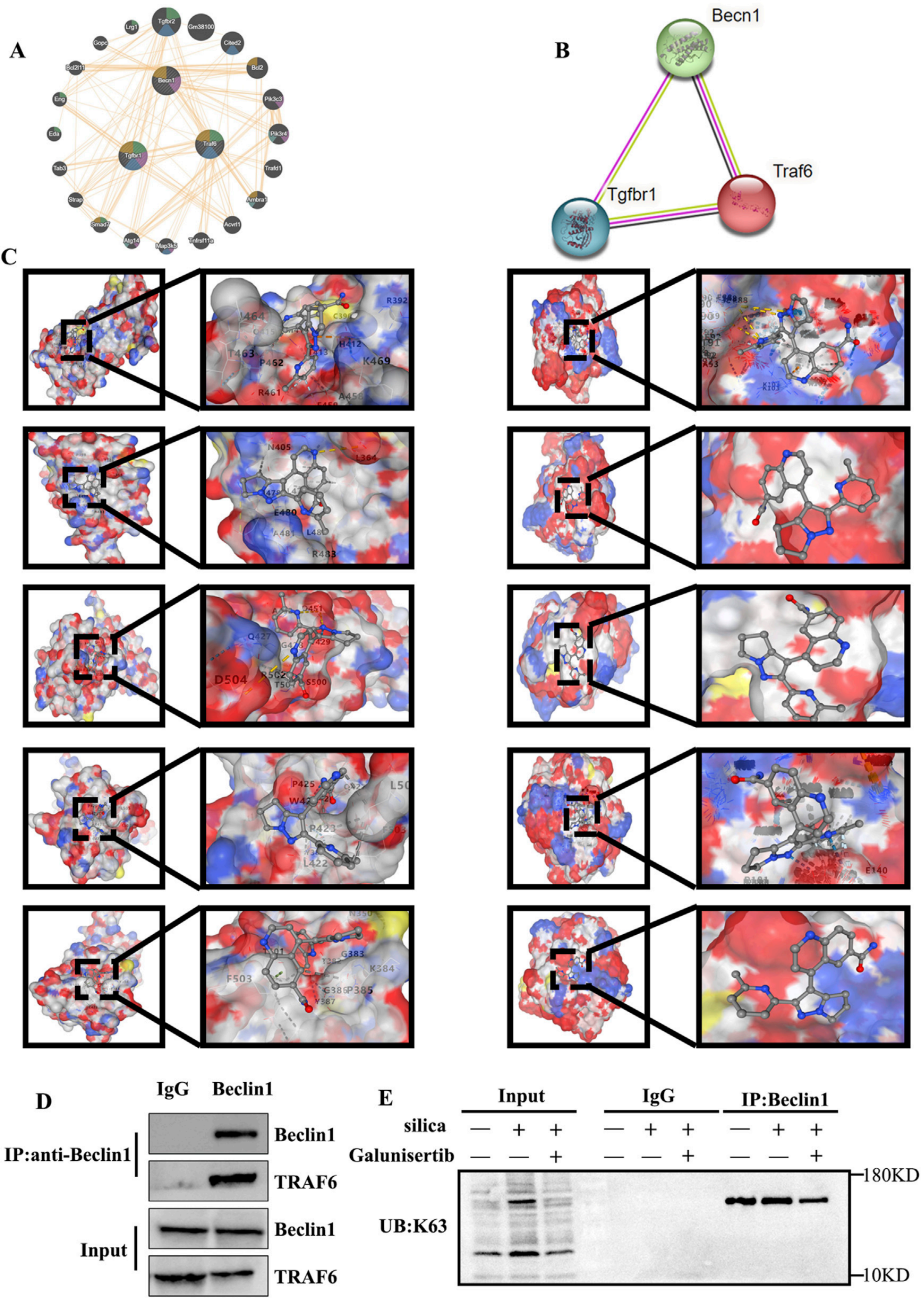
Analysis of potential targets of Galunisertib. **(A,B)** Geneminia and protein-protein network interaction analysis of TGF-β, TRAF6 and Beclin1. **(C)** Molecular docking in TRAF6 (Left) and Beclin1 (Right) with the Galunisertib. **(D)** The interaction between TRAF6 and Beclin1 in mouse lung tissues. **(E)** The levels of K63 ubiquitination in the lung tissues of mice in each group.

To reveal the molecular mechanism involved, we investigated the process of autophagic degradation, which has been considered to be closely associated with apoptotic events. Beclin1 is a key protein involved in the initiation process of autophagy and plays an important role in the process of autophagic degradation. To further explore the mechanism of action of Galunisertib against silicotic fibrosis, we conducted protein-protein interaction analyses using the GENEMANIA and STRING databases and performed co-immunoprecipitation experiments. The results showed that there were interactions among TGF-β, TRAF6, and Beclin1 (Figures 5A,B). Then, Molecular docking simulations were performed between Galunisertib and the target proteins TRAF6, and Beclin1. The binding stability of these complexes is assessed based on the magnitude of the binding scores (Table 1; Figure 5C). Therefore, we speculate that Galunisertib combines with TRAF6 and Beclin1 through TGF-β. Then, we used Co-IP and ubiquitination experiments to detect the binding of TRAF6 and Beclin1 (Figures 5D,E). The results indicated that there was an interaction between TRAF6 and Beclin1. Based on this, the results indicated that there is a close association between Galunisertib and TGF-β/TRAF6/Beclin1 pathway.

**TABLE 1:** The vina scores and the binding pockets.

Target	Compound	Vina score (kcal/mol)	Binding pocket	
			center_x center_y center_z	size_x size_y size_z
TRAF6	Galunisertib	−7.2	19.627 1.345 20.153	21 21 21
		−6.9	5.938 -14.542 -1.899	21 21 21
		−6.1	3.244 2.562 26.810	21 21 21
		−5.9	8.347 6.001 30.944	21 21 21
		−5.9	4.852 11.855 20.258	21 21 21
Beclin1	Galunisertib	−96.1	−8.722 -0.404 -13.731	21 21 21
		−92.5	1.610 -19.008 -7.447	21 21 21
		−83.7	−0.028 -20.399 10.097	21 21 21
		−98.6	−16.448 -8.949 4.983	21 21 21
		−87.6	−17.409 5.359 2.072	21 21 21

### 3.6 Galunisertib inhibits the development of silicosis through the TGF-β/TRAF6/Beclin1 signaling pathway

**FIGURE 6:**
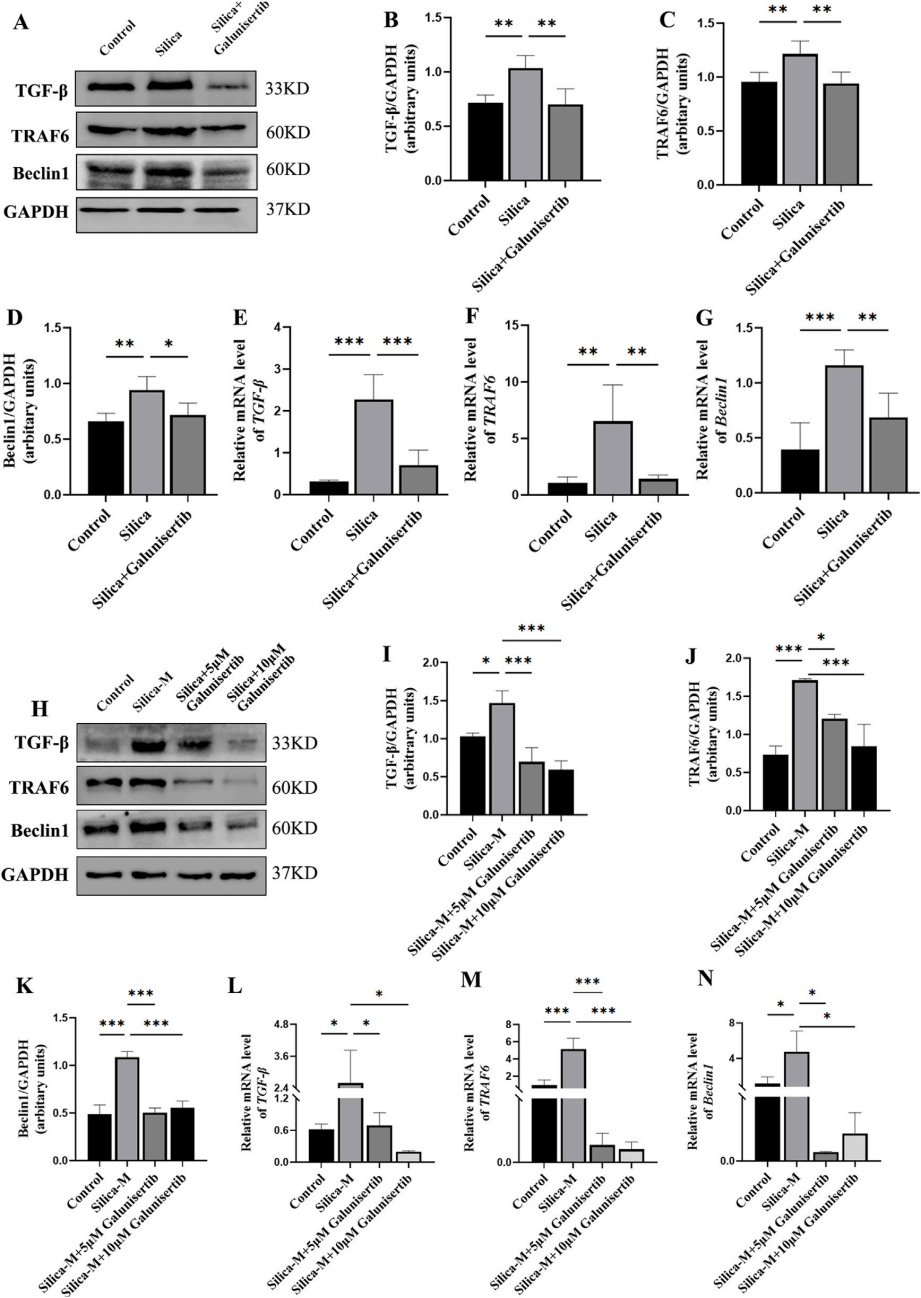
Galunisertib inhibits the development of silicosis through the TGF-β-TRAF6-Beclin1 signaling pathway. **(A–D)** Western blot detection of TGF-β, TRAF6 and Beclin1 protein level in the pulmonary tissue of each group, with GAPDH as the loading control. **(E–G)** qPCR detection of *TGF-β*, *TRAF6* and *Beclin1* transcriptional levels in the pulmonary tissue of each group, with *GAPDH* as the loading control. **(H–K)** Western blot detection of TGF-β, TRAF6 and Beclin1 protein level in fibroblasts of each group, with GAPDH as the loading control. **(L–N)** qPCR detection of *TGF-β*, *TRAF6* and *Beclin1* transcriptional levels in fibroblasts of each group, with *GAPDH* as the loading control. Significance was determined using one-way ANOVA. n = 3–5; ***, *P* < 0.001; **, *P* < 0.01; *, *P* < 0.05.

We further detected the expression levels of TGF-β, TRAF6, and Beclin1 in the lung tissues of mice and fibroblasts to evaluate the mechanism by which Galunisertib affects silicotic pulmonary fibrosis (Figures 5D, 6E–G). The Western blot results showed that, compared with the control group, the protein levels of TGF-β, TRAF6 and Beclin1 in the silica group increased significantly, and the treatment with Galunisertib reduced the expression of TGF-β, TRAF6 and Beclin1 proteins (Figures 6A–D, H–K).

### 3.7 Galunisertib ameliorates autophagic degradation impairment in silicotic mice and silica-stimulated fibroblasts

**FIGURE 7:**
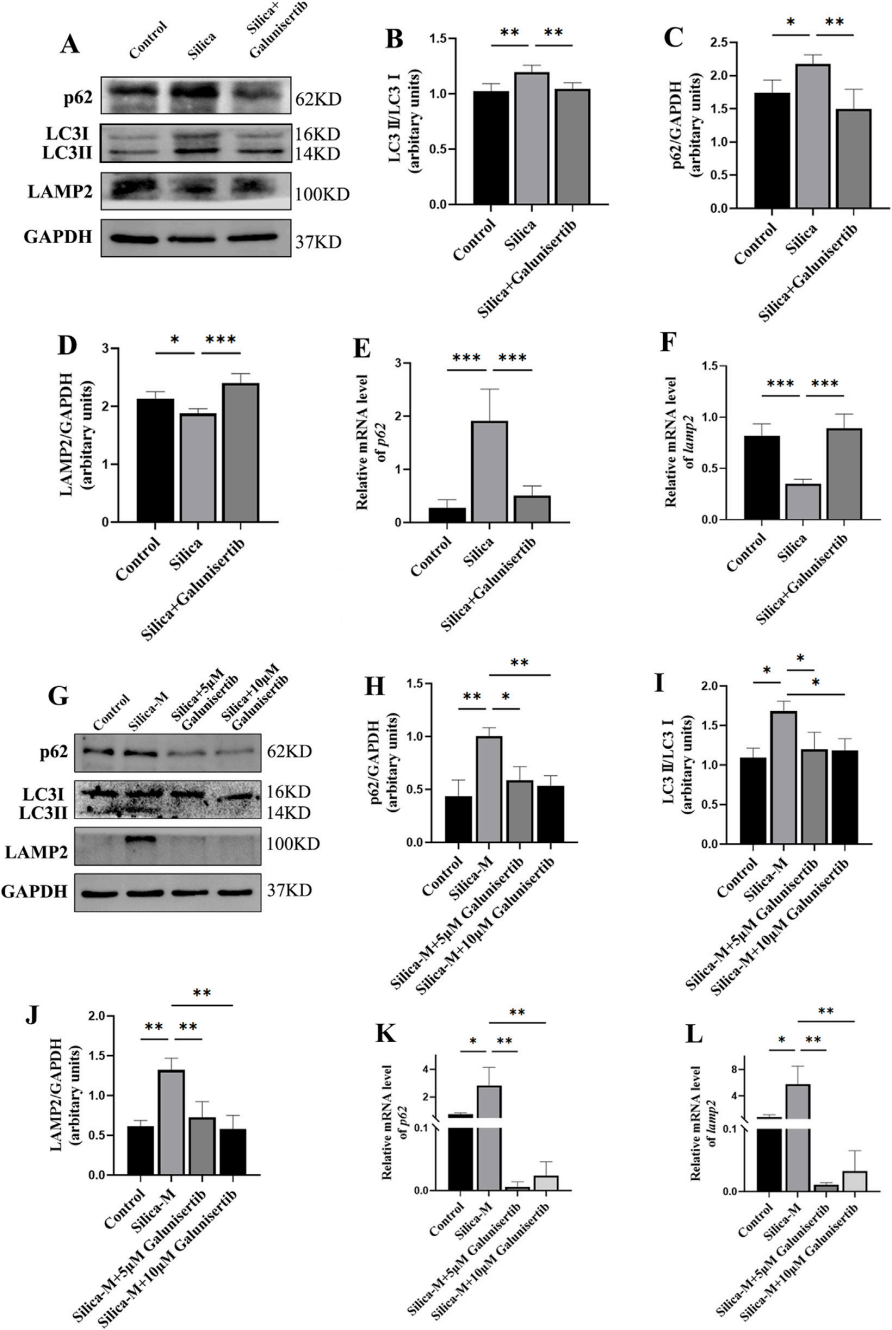
Galunisertib ameliorates autophagic degradation impairment in silicotic mice and Silica-stimulated fibroblasts. **(A–D)** Western blot detection of p62, LC3-II/LC3-I, LAMP2 protein levels in the pulmonary tissue of each group, with GAPDH as the loading control. **(E,F)** qPCR detection of *p62* and *LAMP2* transcriptional levels in the pulmonary tissue of each group, with *GAPDH* as the loading control. **(G–J)** Western blot detection of p62, LC3-II/LC3-I, LAMP2 protein levels in fibroblasts of each group, with GAPDH as the loading control. **(K,L)** qPCR detection of *p62* and *LAMP2* transcriptional levels in fibroblasts of each group, with *GAPDH* as the loading control. Significance was determined using one-way ANOVA. n = 3–5; ***, *P* < 0.001; **, *P* < 0.01; *, *P* < 0.05.

To further validate the specific regulatory effect of Galunisertib on autophagy, we also evaluated the autophagic activity in the lung tissues of mice in each group. Western blot results showed that, compared with the control group, the ratio of LC3Ⅱ/Ⅰ in the lung tissues of mice in the silica dust group increased significantly. After Galunisertib intervention, the ratio of LC3Ⅱ/Ⅰ in the lung tissues of silicotic mice decreased. To further assess the effect of Galunisertib on autophagy in mouse lung tissues, we detected the expression levels of autophagic degradation markers p62 and LAMP2 using Western blot and qPCR. Results showed that, compared with the control group, the protein level of p62 in the lung tissues of mice in the silica dust group increased significantly, while the protein level of LAMP2 decreased significantly. Treatment with Galunisertib reduced the expression of p62 protein and increased the expression of LAMP2 protein (Figures 7A–F). In addition, similar results were also obtained in cell experiments (Figures 7G–L). These results together suggest that Galunisertib can ameliorate autophagic degradation impairment in silicotic mice and fibrosis by inhibiting the TGF-β/TRAF6/Beclin1 signaling pathway.

## 4 Discussion

Silicosis is a preventable occupational lung disease. In recent years, despite the increasing attention to silicosis in various countries, it is still a major public health problem that we need to face (Handra et al., 2023). Silicosis caused by silica dust has a progressive nature, which not only leads to irreversible lung function damage and decreased quality of life, but also significantly increases the social and economic burden due to its irreversibility and long-term treatment requirements. This has become an important challenge in the field of occupational health (Barnes et al., 2019). However, it is regrettable that the current treatment of silicosis still lacks a cure for this disease. In the present investigation, we demonstrated that the therapeutic effects of Galunisertib in mitigating pulmonary fibrosis caused by silica and impeding the transformation of fibroblasts to myofibroblasts. The potential mechanism for protective of Galunisertib in silicosis-related pulmonary fibrosis may be attributed to the inhibition of the TGF-β/TRAF6/Beclin1 signaling pathway, which correlates with ameliorating autophagic flux—evidenced by reduced LC3II/I and p62 accumulation. These findings advocated that Galunisertib might be a valuable therapeutic approach for silicosis fibrosis.

Research has found that activated TGF-β is the central regulatory factor of fibrotic response (Giarratana et al., 2024). The TGF-β signaling pathway not only regulates cell proliferation, differentiation, and apoptosis, but also promotes the transcription of pro-fibrotic factors, leading to epithelial cell death and fibrous tissue proliferation. Additionally, the activation of TGF-β can cause excessive deposition of collagen in tissues, thereby inducing the development of multiple fibrotic diseases (Gifford et al., 2021; Mackinnon et al., 2012). The TGF-β signaling pathway includes downstream canonical (based on Smad) or non-canonical (based on non-Smad) signaling pathways. Activation of the canonical pathway can promote EMT and fibroblast activation, regulate fibroblast proliferation and myofibroblast differentiation, leading to ECM deposition and triggering silicosis fibrosis (Deng et al., 2024; Hu et al., 2018). In addition, TGF-β also enhances and maintains fibrosis response through the expression of PDGF, pro-inflammatory and fibrotic cytokines (Qian et al., 2020). Galunisertib, as a selective inhibitor of TGFβR, has been shown to have anti-inflammatory and anti-tumor effects, and has been applied in clinical trials (Holmgaard et al., 2018). In studies of a cell model for silicosis, treatment with Galunisertib can decrease the expression levels of extracellular matrix while increasing the expression of epithelial phenotype markers (Rong et al., 2015). Our research results also showed that in silicosis mice and fibroblasts treated with silica, the expression levels of TGF-β, fibrosis-related protein Col-I, and α-SMA were significantly upregulated. However, after intervention with Galunisertib, the expression and transcription levels of these proteins were significantly reduced, suggesting that Galunisertib may be a potential drug for treating silicosis fibrosis. Therefore, in our subsequent studies, we administered Galunisertib (300μg/mouse) to silicosis mice to further investigate the mechanism of action of Galunisertib in treating silicosis fibrosis.

The mouse model was divided into two phases: an early inflammatory phase (0–28 days) characterized by lung inflammation, and a fibrotic phase (28–56 days) where fibrosis was the primary pathological feature (Leung et al., 2012; Honnons and Porcher, 2000). Our study demonstrated that both preventative and therapeutic interventions of Galunisertib improved histopathological abnormalities in the silica-induced mouse model and reduced the expression of fibrosis markers such as TGF-β, Col-I, and α-SMA. This effect was specifically evident in the late stage of treatment, suggesting that early recognition and phagocytosis of silica by macrophages may trigger inflammatory cascades involving the release of inflammatory mediators, inflammasome activation, and other inflammation-related pathways, including TGF-β production. Given its role as a TGF-β inhibitor, Galunisertib appears to be particularly important in mitigating fibrosis during the later stages of the disease.

In addition, in non-canonical signaling pathways, the TGF-β receptor complex can interact with TRAF6, promoting TRAF6 ubiquitination and activating downstream signaling pathways such as p38/JNK, NF-κB, and PI3K/AKT/mTOR (Deng et al., 2024; Yamashita et al., 2008). In the above paragraph, TRAF6 can interact with Beclin1 and induce ubiquitination of Beclin1, leading to autophagy induction (Shi and Kehrl, 2010; Kim et al., 2022). In recent years, autophagy, as a critical programmed cell death mechanism, has been confirmed to be involved in various disease processes (Debnath, Gammoh, et al., 2023; Tan and Chen, 2021). Frequent exposure to various external stimuli can affect the level of autophagy in the human lungs. Autophagy maintains cell function by degrading abnormal substances, thereby regulating the pathological process of lung diseases (Chen et al., 2018). At the same time, autophagy is a regulator of fibrosis, which can inhibit collagen deposition and EMT in fibroblasts, thereby inhibiting the fibrosis process (Siapoush et al., 2023). To further investigate the role of Galunisertib in inhibiting TGF-β in anti-silicosis fibrosis, we used the GENEMANIA and STRING databases to analyze the interactions between TGF-β, TRAF6, and Beclin1. Our results showed that Galunisertib inhibits the expression of TRAF6 by inhibiting TGF-β, and there is a significant interaction between TRAF6 and Beclin1. Therefore, we infer that Galunisertib may regulate autophagy through the TGF-β/TRAF6/Beclin1 signaling pathway.

During the process of autophagy, LC3 acts as a key protein in the formation of autophagosomes. It can be lipidated from LC3-I to LC3-II and specifically involved in the closure of autophagosomes (Li et al., 2021; Chen et al., 2024). As a selective autophagy receptor, p62 targets waste materials for transport to autophagosomes. Under normal physiological conditions, LC3 and p62 are degraded during autophagy and do not accumulate in cells (Walter et al., 2023). LAMP2 mainly participates in the fusion of autophagosomes and lysosomes (Zhang et al., 2023). These three proteins work together to ensure the smooth completion of the autophagy process (Wei et al., 2024). In our previous studies on AMs in silicosis patients and in a silicosis mouse model, we observed changes in the level and function of autophagy, characterized by a decrease in lysosomes and an increase in autophagosomes, as well as an increase in the autophagic degradation markers LC3II/I and p62, and a decrease in LAMP2 expression (He et al., 2020; Tan et al., 2023). However, some researchers have also pointed out that there may be chaperone-mediated autophagy in synovial fibroblasts, which does not rely on the formation of autophagosomes, but rather on the co-expression of chaperone protein Hsc70 and LAMP2A. When the level of inflammation increases, the level of LAMP2 in synovial fibroblasts significantly increases (Fu et al., 2022). Therefore, we further investigated the effect of Galunisertib on autophagic degradation function in the lung tissue of silicosis mice. WB and qPCR analysis revealed that Galunisertib reversed the upregulation of LC3II/I, p62, and downregulation of LAMP2 in the lung tissue of silicosis mice, indicating that the accumulation of autophagic substrates and impairment of lysosomal function in the lung tissue of silicosis mice were improved by Galunisertib intervention, which is consistent with our previous research results (Tan et al., 2023; Tan et al., 2020a). However, the level of LAMP2 in silica-stimulated mouse fibroblasts showed a completely opposite trend compared to that in the lung tissue of silicosis mice. The opposite trend may be due to the continuous presence of silica in the lungs of silicosis mice, which may lead to obstruction of the autophagy pathway. This results in a significant downregulation of LAMP2 levels. However, in fibroblasts stimulated with supernatant, the autophagy pathway may be activated by inflammatory factors and accompanied by chaperone-mediated autophagy, leading to an upregulation of LAMP2 levels.

In addition, when TGF-β binds to TGF-β receptors to form a complex, it can also activate and phosphorylate Transforming Growth Factor-β-Activated Kinase 1 (TAK1), which then phosphorylates JNK and p38, initiating the downstream signaling pathways that lead to apoptosis (Rong et al., 2015; Zhou et al., 2018; Aashaq et al., 2019). Therefore, we further investigated the effect of Galunisertib on apoptosis. WB and qPCR results showed that, compared to the silicosis model group, the Galunisertib intervention group had decreased expression levels of the apoptosis execution protein Cleaved-caspase 3 in lung tissue. This suggests that Galunisertib may regulate the autophagy-lysosomal system through the TGF-β/TRAF6/Beclin1 signaling pathway, thereby reducing cell apoptosis and improving fibrosis in silicosis mice.

Silicosis progresses through distinct inflammatory and fibrotic stages, with alveolar macrophages and fibroblasts serving as key mediators throughout this process. Thus, in subsequent studies, we aim to conduct in-depth investigations into the specific role of macrophages in silica-induced pathological progression. Notably, this study only utilized male mice, a factor that may restrict the generalizability of our findings. Therefore, future research should focus on exploring the specific regulatory mechanisms of macrophages, while also validating our results in female mice to further complement and enhance the comprehensiveness of the current study.

## 5 Conclusion

In this study, we revealed that Galunisertib can modulate autophagy in pulmonary tissues of silicotic mice and fibroblast cells by suppressing the TGF-β/TRAF6/Beclin1 signaling pathway. On the other hand, Galunisertib regulates autophagy and inhibits the activation, proliferation and migration of Silica-stimulated fibroblasts, alleviating fibrosis in silicosis mice. Therefore, Galunisertib may be a promising drug for the treatment of silicosis and warrants further investigation.

## Data Availability

The datasets presented in this study can be found in online repositories. The names of the repository/repositories and accession number(s) can be found in the article/Supplementary Material.

## Supplementary material

SUPPLEMENTARY FIGURE S1 Flow chart of the experimental protocol. **(A)** The mice were randomly divided into the following four treatment groups:(1) Control (Control) group, mice without any treatment; (2) crystalline silica (Silica) group, mice received intratracheal injection of crystalline CS suspended in saline; (3) Galunisertib low dose group, mice received 100 µg/animal Galunisertib i.p.; and (4) Galunisertib high dose group, mice received 300 µg/animal Galunisertib i.p. Mouse lungs were collected 56 days after CS and/or Galunisertib administration. **(B)** The mice were randomly divided into the following three treatment groups: (1) Control (Control) group, mice without any treatment; (2) crystalline silica (Silica) group, mice received intratracheal injection of crystalline CS suspended in saline; (3) Galunisertib (Silica + Galunisertib) group, mice received 300 µg/animal Galunisertib i.p.; Mouse lungs were collected 28 days after CS and/or Galunisertib administration. **(C)** The mice were randomly divided into the following three treatment groups: (1) Control (Control) group, mice without any treatment; (2) crystalline silica (Silica) group, mice received intratracheal injection of crystalline CS suspended in saline; (3) Galunisertib (Silica + Galunisertib) group, mice received 300 µg/animal Galunisertib **(I)**p.; Mouse lungs were collected 56 days after CS and/or Galunisertib administration.

SUPPLEMENTARY FIGURE S2 Galunisertib delayed silica-induced pulmonary fibrosis progression *in vivo* in preventive therapeutic intervention regimens. **(A)** Body weights of experimental mice from indicated groups. **(B)** Representative images of lung tissue from each group of mice. **(C)** H&E and Masson staining of the mouse lung tissue (original magnification: upper images, ×50 and lower images, ×100). **(D)** The score analysis of Masson staining in the mouse lung tissue. **(E)** Quantification of collagen in the mouse lung tissue by HYP content. **(F)** Lung weights of mice in each group. **(G)** The pulmonary index was determined to show the alveolar swelling and interstitial fibrosis in mice. **(H–K)** Western blot detection of TGF-β, Col-Ⅰ and α-SMA of each group, with GAPDH as the loading control. Significance was determined using one way ANOVA. n = 3–5; The right arrows represent inflammatory cell infiltration and damaged alveolar structure, and the down arrows represent collagen deposition. ***, *P* < 0.001; **, *P* < 0.01; *, *P* < 0.05.

## Abbreviations

AMs, Alveolar macrophages; Col, Collagen; GAPDH, Glyceraldehyde-3-phosphate dehydrogenase; LC3, The receptor protein microtubule-associated protein 1 light chain 3; p62/SQSTM1, p62/sequestosome1; α-SMA, Alpha-smooth muscle actin; ECM, Extracellular Matrix; HE, Hematoxylin and eosin; HYP, Hydroxyproline; LAMP2, Lysosome-associated membrane protein 2; mRNA, Messenger Ribonucleic Acid; TGF-β, Transforming Growth Factor-β; TRAF6, Tumor necrosis factor receptor-associated factor 6; TAK1, Transforming Growth Factor-β-Activated Kinase 1.
