# Supplementary figures and images for: Galunisertib attenuates pulmonary fibrosis with silicosis in mouse via TGF-β/TRAF6/Beclin1 signaling pathway
Source: Front Pharmacol. 2025 Nov 25;16:1702511. doi: 10.3389/fphar.2025.1702511 (PMC12685898; doi:10.3389/fphar.2025.1702511)

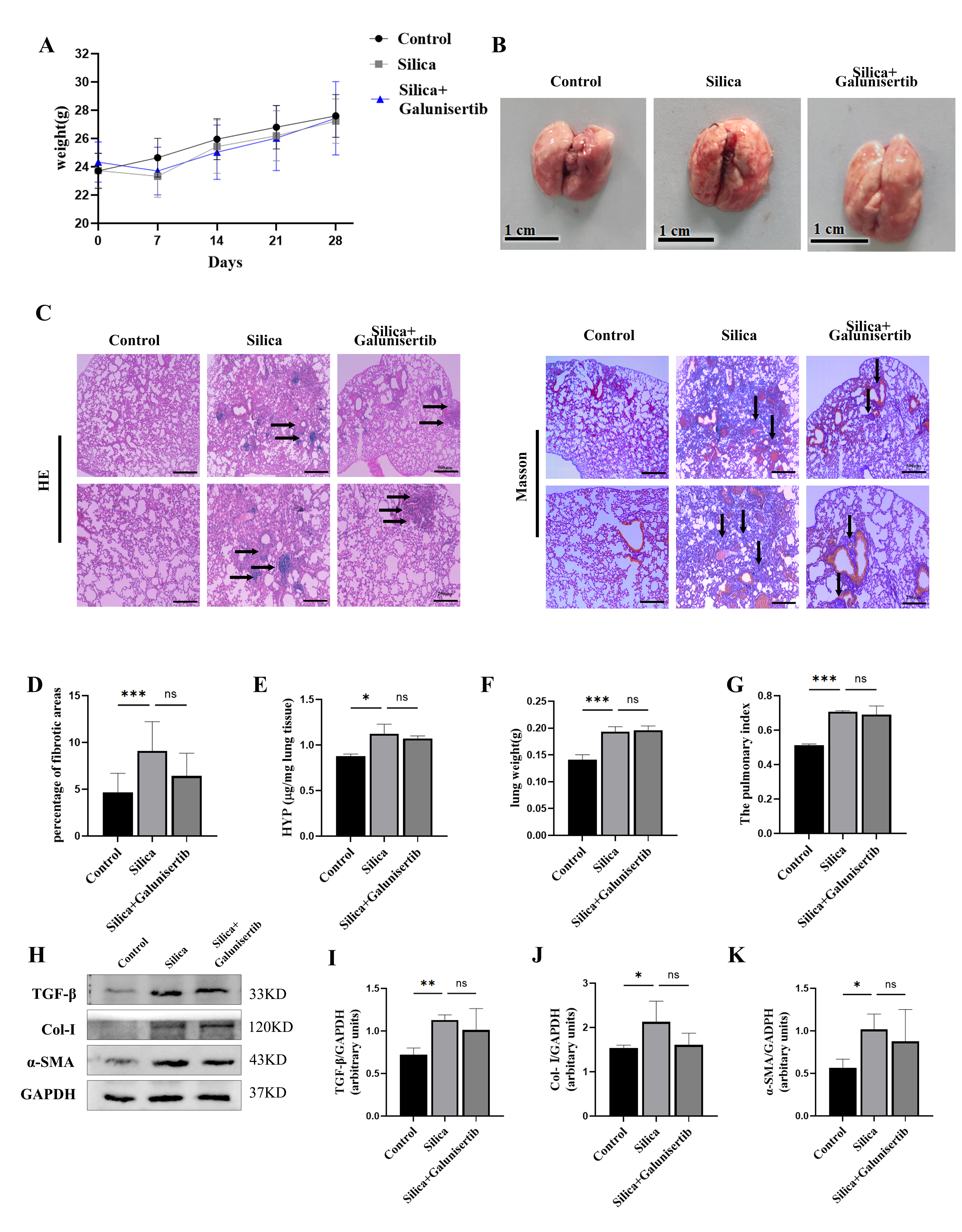

Supplement: Supplementary file 2 [file Image2.tif]

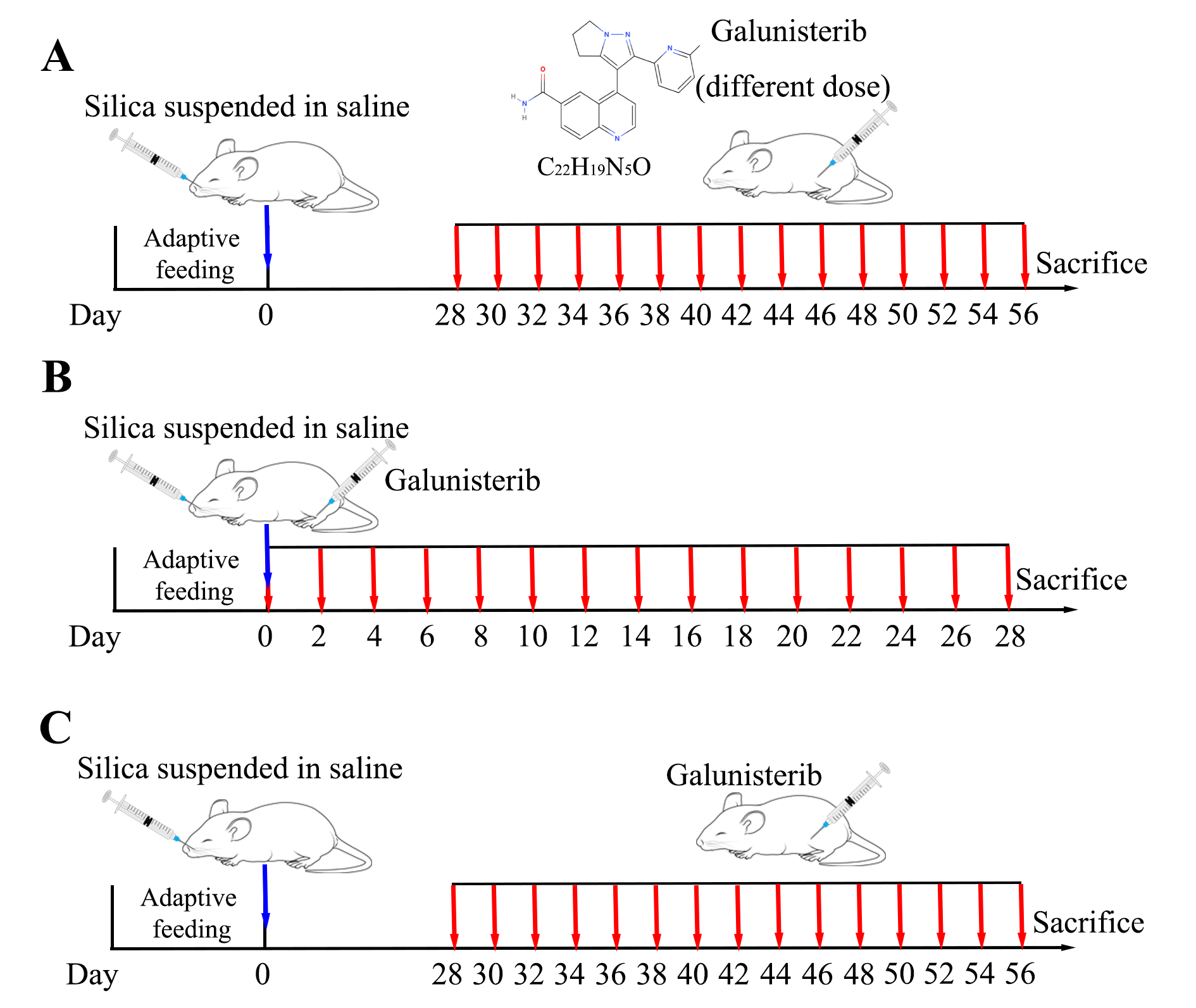

Supplement: Supplementary file 3 [file Image1.tif]
